# Langerhans Cells as Morphologic Mimickers of Atypical Melanocytes on Reflectance Confocal Microscopy: A Case Report and Review of the Literature

**DOI:** 10.5826/dpc.1103a78

**Published:** 2021-05-20

**Authors:** Nadiya Chuchvara, Lauren Berger, Catherine Reilly, Amin Maghari, Babar K. Rao

**Affiliations:** 1 Center for Dermatology, Rutgers Robert Wood Johnson Medical School, Somerset, New Jersey, US; 2 Department of Pathology and Laboratory Medicine, Rutgers Robert Wood Johnson Medical School, New Brunswick, New Jersey, US; 3 Department of Dermatology, Weill Cornell Medicine, New York, New York, US

**Keywords:** reflectance confocal microscopy, RCM, Langerhans cells, dendritic cells, atypical cells

## Abstract

Pagetoid spread of melanocytes in the epidermis is a common indicator of melanocytic atypia, both histopathologically and with reflectance confocal microscopy (RCM). Specifically on RCM, large, bright, atypical dendritic and/or roundish cells are characteristic of melanoma. However, intraepidermal Langerhans cells (ILC) create the potential for diagnostic ambiguity on RCM. We describe one case of a pigmented facial lesion that was initially diagnosed as lentigo maligna (LM) due to numerous atypical perifollicular dendritic cells on RCM. Additionally, we present the findings of a literature review for similar reported cases conducted by searching the following terms on PubMed: reflectance confocal microscopy, RCM, lentigo maligna, melanoma, Langerhans cells, dendritic cells, and atypical cells. In our case, the lesion was determined to be a solar lentigo on histopathology. Immunohistochemistry (IHC) with CD1a identified the atypical-appearing cells as ILC, as it did in 54 reported cases of benign lesions (benign melanocytic nevus, Sutton/halo nevus, labial melanotic macule, and solar lentigo) misdiagnosed as malignant on RCM (melanoma, lip melanoma, lentigo maligna, and LM melanoma). According to our case and the literature, both ILC and atypical melanocytes can present with atypical-appearing dendritic and/or roundish cells under RCM. Currently, there is no method to distinguish the two without IHC. Therefore, the presence of pagetoid cells should continue to alert the confocalist of a potential neoplastic process, prompting biopsy, histopathologic diagnosis, and IHC differentiation.

## Introduction

Reflectance confocal microscopy (RCM) is a technique that acquires *en face* images of the epidermis and papillary dermis in vivo, using a non-invasive laser device (830 nm). Confocal images have a resolution comparable to traditional histopathology [1]. This enables high accuracy diagnosis without the use of biopsy, particularly for pigmented lesions, in which melanin provides strong endogenous contrast [2]. Melanocytic cytologic atypia is suggested by the presence of large (>20 μm), bright, dendritic, or roundish cells [3]. Pagetoid melanocytosis observed in RCM has been histopathologically correlated to melanoma [4–7]. While not specific for malignancy, the presence of pagetoid spread on RCM of a pigmented lesion carries 11 to 22 [7,8] times greater risk of melanoma, with odds ratios of 4 to 9 for dendritic cells [4,6,8] and 9.7 to 108 [4–6,8] for roundish cells.

Suspicion for melanoma on RCM increases when atypical cells are densely distributed (>5 cells/mm^2^), pleomorphic with large and unusual morphology (triangular, star-shaped), roundish, diffuse, and extend to the stratum corneum [3,5,6]. Atypical dendritic or roundish pagetoid cells with folliculotropism are characteristic of lentigo maligna (LM) [9,10]. We describe a case in which a benign pigmented lesion on the cheek resembled LM on confocal images, owing to dendritic intraepidermal Langerhans cells (ILC) misinterpreted as atypical melanocytes. We present a literature review of additional cases in which the presence of ILC resulted in erroneous diagnosis of melanoma on RCM.

### Case Report

**Figure 1:**
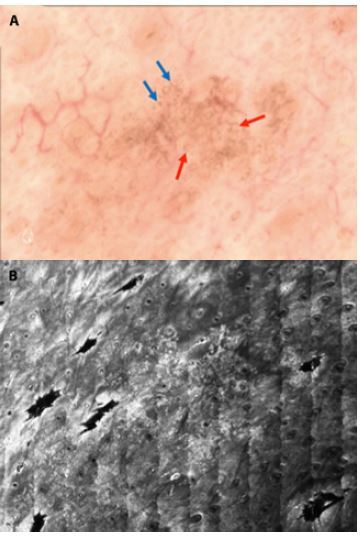
(A) Dermoscopic image of the 5 mm light brown papule, showing blue-gray granularity (blue arrows) and crescent-shaped perifollicular pigmentation (red arrows). (B) Corresponding RCM mosaic image at the level of the epidermis, acquired using the VivaScope 1500 (Caliber I.D., Rochester, NY) reflectance confocal microscope.

A 64-year-old Caucasian woman presented with a 5 mm light brown papule on the left cheek that had been present for several months and was growing in size. She had a history of blistering sunburns in childhood and basal cell carcinoma of the right hand 13 years prior to presentation. Family history was significant for ocular melanoma in the patient’s mother. Dermoscopic examination of the lesion showed blue-gray granularity and crescent-shaped perifollicular pigmentation, which are considered indicative of melanophages in the dermis and atypical melanocytes extending down hair follicles (Figure 1A) [11,12]. The patient was referred for RCM due to provider suspicion for malignancy and patient preference for a non-invasive procedure (Figure 1B).

**Figure 2:**
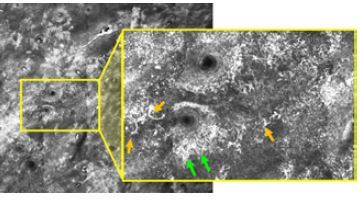
RCM image taken at the level of the epidermis. Inset demonstrates large, atypical pagetoid dendritic cells (orange arrows), with many in a perifollicular distribution (green arrows).

**Figure 3:**
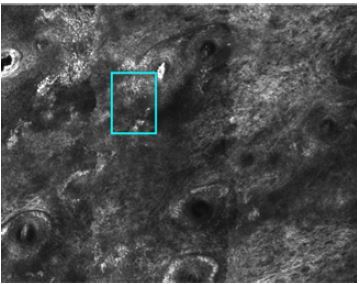
RCM image taken at the level of the DEJ, with focal areas of small bright and large bright inflammatory cells (blue box).

RCM revealed an irregular honeycomb pattern with numerous large (>20 μm) atypical dendritic pagetoid cells, including some in a perifollicular distribution, consistent with LM (Figure 2). The dermo-epidermal junction (DEJ) contained focal areas of small bright and large bright inflammatory cells (Figure 3).

**Figure 4:**
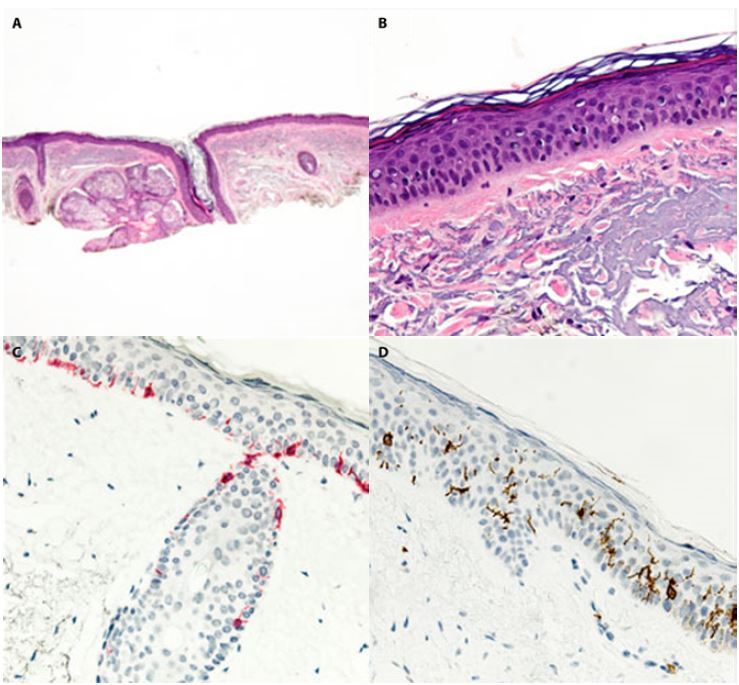
(A) SL with underlying solar elastosis and sebaceous hyperplasia (H&E, x40). (B) Absence of atypical or dendritic cells with standard H&E (x200). (C) IHC with Melan-A showing a normal distribution of benign-appearing melanocytes in the epidermis, consistent with SL (x200). (D) IHC with CD1a revealing numerous Langerhans cells throughout the epidermis (x200)

A shave biopsy was performed, and tissue sections were stained with hematoxylin and eosin (H&E) (Figure 4A, B). Histopathologic analysis revealed solar lentigo (SL) with underlying sebaceous hyperplasia. Immunohistochemistry (IHC) staining for the melanocyte-specific Melan-A showed a normal distribution of benign-appearing melanocytes in the epidermis, consistent with SL (Figure 4C). Staining for CD1a, a membrane glycoprotein specific for Langerhans cells and immature T cells, revealed numerous Langerhans cells throughout the epidermis (Figure 4D). Thus, the atypical dendritic cells visualized on RCM likely represented ILC, rather than atypical melanocytes.

## Discussion

RCM is an accurate tool for non-invasive differentiation between benign and malignant melanocytic lesions. For the diagnosis of LM specifically, Guitera et al [10] isolated characteristic RCM features to develop an algorithmic “LM score,” resulting in a sensitivity of 85% and specificity of 76% for lesions with scores ≥2. Two major features earn +2 points each (nonedged papillae and large round pagetoid cells >20 μm), three minor features earn +1 point each (three or more atypical cells at the DEJ in five images, follicular localization of atypical cells, and nucleated cells within the papilla), and one minor feature earns −1 point (broadened honeycomb pattern) [10]. Furthermore, Gomez-Martin et al [13] demonstrated the utility of RCM in the diagnosis of ambiguous pigmented facial macules (91.7% sensitivity and 86.8% specificity for LM). They found two dermoscopic features (asymmetric follicular pigmentation and target like structures) and two RCM features (round, large pagetoid cells and follicular localization of atypical cells) to be associated with LM/LMM [13].

In this case, the diagnosis was compromised due to shared morphologic features of Langerhans cells and atypical melanocytes on RCM [14]. Both cell types tend to appear as bright cells in a pagetoid pattern on RCM, often with a dendritic morphology [5]. This explains why intraepidermal dendritic cells can be found in both LM/lentigo maligna melanoma (LMM) and benign pigmented facial macules [13]. The bright appearance of Langerhans cells is likely due to their Birbeck granules, which have a high reflection index and thus appear light gray to white under RCM, similar to melanin [14]. Langerhans cells are normally present in the epidermis and serve as antigen-presenting cells for T lymphocytes [15]. Among benign lesions, they are more likely to be prominent around inflammation, such as traumatized benign melanocytic nevi (BMN), recent scars, or lichen planus-like keratosis (LPLK) [16,17]. When identified on RCM in high densities, ILC are more likely to result in a false diagnosis of melanoma [14]. While dendritic cells alone are not enough to diagnose LM, as demonstrated by Guitera et al [10] and Gomez-Martin et al [13], the follicular localization of atypical cells on RCM, correlating to asymmetric follicular pigmentation on dermoscopy, raised the level of suspicion for LM in our patient’s case.

In the literature, ILC presence has been confirmed in several cases of benign lesions that were perceived to be malignant under RCM. These include suspected cases of melanoma [14,18,19], lip melanoma [20], and LM/LMM [13] (Table 1).

Comparatively high densities of Langerhans cells exist within the head, neck, trunk, and limbs [21], corresponding to the variety of locations reported. Most cases described RCM findings of roundish and dendritic pagetoid cells, although some cases found atypical cells at the DEJ and dermis, as well. As evidenced by our case, traditional H&E stain was not sufficient in identifying ILC on histopathology and CD1a was required for further classification. In some studies, dendritic pagetoid cells were identified by RCM in lesions that were ultimately benign, but staining for Langerhans cells was not pursued [10,22,23]. Thus, the prevalence of ILC in pigmented lesions, and its confounding effect on RCM diagnosis, may be even greater than reported. The inability to use special stains and IHC when conducting in vivo RCM makes such morphologic mimickers indistinguishable across several lesion types.

Some malignant and pre-malignant lesions, particularly pigmented basal cell carcinomas [17,24], in situ or early invasive melanomas [14,25,26], pigmented actinic keratosis [27], and pigmented squamous cell carcinoma (SCC) in situ [28], may also contain a high density of Langerhans cells. In a study classifying melanoma into distinct types, Pellacani et al [29] found that in situ and thin melanomas (<1 mm Breslow thickness) were characterized by dendritic cells on RCM. While the authors did not further identify the cells immunohistochemically to rule out the possibility of ILC, this study, and others like it, highlight a possible association of dendritic cells with thin, or early, melanomas. In cases of larger suspicious pigmented lesions, RCM has the added benefit of real-time biopsy guidance, increasing the sensitivity for histopathologic detection of malignancy [10]. Future studies would need to determine whether the identification of dendritic cells on RCM of a clinically ambiguous lesion would result in earlier detection of melanoma.

While atypical cells have been identified as ILC rather than atypical melanocytes across benign lesions including BMN, Sutton (halo) nevi, labial melanotic macules, and SL, the presence of dendritic pagetoid cells should continue to alert the confocalist of a potential neoplastic process, prompting biopsy, histopathologic diagnosis, and IHC differentiation.

## Figures and Tables

**Table 1:** Reported cases of Langerhans cells presenting as atypical melanocytes in benign lesions on RCM.

RCM Diagnosis	Reference	Case Composition	Clinical Features	RCM Cytologic Findings	Histopathologic
Melanoma	Hashemi et al.^14^	24 cases of BMN falsely diagnosed as melanoma	Pigmented lesions in various locations including shoulder, back, abdomen (others unspecified)	Bright cells in a pagetoid pattern: 5/24 roundish, 4/24 dendritic, 15/24 both	Analysis BMN, CD1a positive, 7/24 Melan-A positive, cytokeratin-20† negative
	Yelamos et al.^18^	1 case of recurrent nevus falsely diagnosed as melanoma	Pigmented macule on right knee	Pleomorphic, mostly dendritic cells throughout the epidermis, DEJ, and in the dermal nests	CMN with fibrosis suggestive of recurrent nevus; CD1a positive, SOX10 normal
	Brugues et al.^19^	21 cases of clinically atypical Sutton (halo) nevi excised due to possibility of melanoma	Pigmented macules with atypical dermoscopic features such as: asymmetrical peripheral whitish halo, white/blue-gray regression, peppering	13/21 atypical pagetoid cells (dendritic > roundish); atypical basal cells (roundish = dendritic); dermal atypical nucleated cells, plump cells, and bright particles	Sutton nevus, CD1a positive, Melan-A positive (large melanocytes) in the epidermis and DEJ
Lip melanoma	Porto et al.^20^	3 cases of labial melanotic macule falsely diagnosed as lip melanoma	Pigmented macules on the lower lip	3/3 bright dendritic cells at the DEJ, around and between dermal papillae	Labial melanotic macule, CD1a positive, Melan-A and S-100 negative (1/3) or normal (2/3)
LM/LMM	Gomez-Martin et al.^13^	5 cases of pigmented facial macules falsely diagnosed as LM/ LMM	Clinically ambiguous pigmented facial macules	5/5 abundant dendritic pagetoid cells; 4/5 round, large pagetoid cells; 3/5 atypical cells at the DEJ	CD1a positive, 2/5 showed both basal melanocyte hyperplasia and ILC secondary to postradiotherapy pigmentation
	Current case	1 case of SL falsely diagnosed as LM	Pigmented facial papule with dermoscopic features concerning for LM	Large, atypical dendritic pagetoid cells in a perifollicular distribution	SL with underlying nodular sebaceous hyperplasia, CD1a positive, Melan-A normal

BMN= benign melanocytic nevus/nevi; CMN = compound melanocytic nevus; DEJ = dermo-epidermal junction; ILC= intraepidermal Langerhans cell(s); LM = lentigo maligna; LMM – lentigo maligna melanoma; RCM = reflectance confocal microscopy; SL= solar lentigo

† marker for Merkel cells
